# Genome Instability-Associated Long Non-Coding RNAs Reveal Biomarkers for Glioma Immunotherapy and Prognosis

**DOI:** 10.3389/fgene.2022.850888

**Published:** 2022-04-27

**Authors:** Xinzhuang Wang, Hong Zhang, Junyi Ye, Ming Gao, Qiuyi Jiang, Tingting Zhao, Shengtao Wang, Wenbin Mao, Kaili Wang, Qi Wang, Xin Chen, Xu Hou, Dayong Han

**Affiliations:** ^1^ Department of Neurosurgery, The First Affiliated Hospital of Zhengzhou University, Zhengzhou, China; ^2^ Department of Hematology, Liaocheng People’s Hospital, Liaocheng, China; ^3^ Department of Neurosurgery, First Affiliated Hospital of Harbin Medical University, Harbin, China; ^4^ Biochip Laboratory, Yantai Yu-Huang-Ding Hospital, Qingdao University, Yantai, China; ^5^ The First Affiliated Hospital of Harbin Medical University, Harbin, China

**Keywords:** glioma, genome instability, lncRNA, tumor microenvironment, MDNAsi, TCGA

## Abstract

Genome instability is a hallmark of tumors and is involved in proliferation, invasion, migration, and treatment resistance of many tumors. However, the relationship of genome instability with gliomas remains unclear. Here, we constructed genome instability-derived long non-coding RNA (lncRNA)-based gene signatures (GILncSig) using genome instability-related lncRNAs derived from somatic mutations. Multiple platforms were used to confirm that the GILncSig were closely related to patient prognosis and clinical characteristics. We found that GILncSig, the glioma microenvironment, and glioma cell DNA methylation-based stemness index (mDNAsi) interacted with each other to form a complex regulatory network. In summary, this study confirmed that GILncSig was an independent prognostic indicator for patients, distinguished high-risk and low-risk groups, and affected immune-cell infiltration and tumor-cell stemness indicators (mDNAsi) in the tumor microenvironment, resulting in tumor heterogeneity and immunotherapy resistance. GILncSig are expected to provide new molecular targets for the clinical treatment of patients with gliomas.

## Introduction

Glioma is the most common primary central nervous system tumor in adults and accounts for approximately 80.8% of all malignant central nervous system tumors (Ostrom et al., 2020). Mutations in epigenetic regulatory genes contribute to the formation of different subtypes of gliomas, resulting in very limited effects of conventional treatments (Ostrom et al., 2014; Berghoff et al., 2017). Among them, glioblastoma is the most malignant type of glioma and has the worst prognosis. Even with standardized treatment, the median survival time remains less than 2 years (Stupp et al., 2009; Tan et al., 2020). Patients with functional-area or end-stage tumors often exhibit neurological symptoms caused by tumor space-occupying effects, which may seriously affect the quality of life and health of patients (Lapointe et al., 2018). In recent years, with the rapid development of genomics, there has been a greater understanding of the pathogenesis of glioma (Berger and Mardis, 2018). Some new treatments have been applied clinically, but most gliomas remain insensitive or tolerant to these treatments (Nduom et al., 2015; Jackson et al., 2019). This may be due to a lack effective molecular targets (Lee et al., 2018). Therefore, to improve the prognosis of patients and guide molecular therapy for clinical glioma, there is a need to identify new molecular markers.

Genome instability is an important factor for genome diversity and natural selection and is also a basic characteristic of tumor formation (Negrini et al., 2010; Tubbs and Nussenzweig, 2017). Moreover, genetic instability is widely involved in the occurrence and development of tumors and is related to patient prognosis (Ottini et al., 2006). For example, Koschmann et al. found that ATRX loss can increase glial genome instability and promote tumor progression (Koschmann et al., 2016). Zhang et al. found that high expression of the gene module co-expressed with CDC20 is closely related to chromosome instability and may be a potential molecular target for the treatment of glioma (Zhang et al., 2019). In recent years, some long non-coding RNAs (lncRNAs) have also been found to be closely related to genome instability and tumor progression. For example, Zho et al. found that gene instability-related lncRNAs are widely involved in breast cancer gene instability through cell cycle arrest and that they suggest a poor prognosis for patients (Bao et al., 2020). Mathias et al. found that the lncRNA noncoding RNA activated by DNA damage (NORAD) can be combined with the RNA-binding motif protein encoded on the X chromosome (RBMX) to maintain gene stability by forming a topoisomerase complex (Munschauer et al., 2018). However, the relationship between genetic instability and lncRNAs in gliomas is still unclear.

The tumor microenvironment is a complex composed of immune cells, stromal cells, extracellular matrix, and tumor cells (Cui et al., 2017). During malignant progression of tumor cells, the cells gradually dedifferentiate and acquire characteristics of stem cells. Accordingly, the tumor immune microenvironment and tumor stem cells are important components of tumor proliferation, metastasis, and treatment resistance (Pitt et al., 2016; Malta et al., 2018). Studies have also found that cancer stem cell-like characteristics can affect the mutation status of oncogenes and tumor suppressor genes, thereby increasing genome instability (Zhang et al., 2020). Genome instability leads to increased tumor mutation burden, which makes it easier for immune cells to recognize and promote immune cell infiltration, reduce tumor purity, and increase tumor tissue heterogeneity (Mouw et al., 2017; Zhang et al., 2017). In view of the intricate relationship among genome instability, tumor microenvironment, and tumor cell stemness, understanding the underlying pathogenesis may help develop new strategies for tumor treatment. Therefore, it is particularly important to investigate the mechanisms related to glioma genome instability (Sansregret et al., 2018).

In the current study, we constructed a genome stability and gene instability framework based on somatic mutation data of glioma and found 23 lncRNAs related to genome instability. We then identified seven genome instability-derived lncRNA-based gene signatures (GILncSig). Multiple platforms were used to verify that the GILncSig were closely related to patient prognosis and clinical characteristics. We also analyzed the relationship between the GILncSig, glioma microenvironment, and glioma cell stemness index to help develop new strategies for the clinical treatment of patients with glioma. Through laboratory-based experiments and analysis of data from the TICA and pRRophetic databases, we showed that patients in the high-risk group were relatively insensitive to immunotherapy but were relatively sensitive to treatment with cisplatin and rapamycin. GILncSig are expected to provide new molecular targets for the clinical treatment of patients with gliomas.

## Materials and Methods

### Data Extraction

Clinical characteristics, gene expression matrix, and somatic mutation information of 698 glioma patients were extracted from The Cancer Genome Atlas (TCGA) database (https://portal.gdc.cancer.gov/). Furthermore, clinical information and data on the lncRNA expression levels of 693 glioma specimens were extracted from the Chinese Glioma Genome Atlas (CGGA) mRNAseq_693 database and 325 glioma specimens from the CGGA mRNAseq_325 database (http://www.cgga.org.cn/) (Bao et al., 2014; Wang et al., 2015). Information on the stemness indicators of glioma cells, including the stemness index based on mRNA expression (mRNAsi), DNA methylation-based stemness index (mDNAsi), and epidermal growth factor receptor messenger RNA expression (EGFR-mRNA), was obtained from published articles (Pan et al., 2019).

### Genome Instability-Related lncRNAs From Somatic Mutations

Based on TCGA data, we calcul1ated the frequency of somatic mutations in each of the glioma specimens and sorted them in a descending order. The first 25% of the specimens were considered to be genome unstable (GU)-like samples and the last 25% were considered to be genome stable (GS)-like samples. In total, 23 lncRNAs were identified via screening significance analysis of microarrays according to log-fold change (logFC) expression differences (logFC >3 or logFC < −3; *p* < 0.001) (Bao et al., 2020).

### Construction of the GILncSig Model

All the glioma samples were randomly assigned to two cohorts—332 samples in the test group and 335 samples in the training group. Seven lncRNAs related to prognosis were screened from the training group through univariate and multivariate Cox regression analyses, and a prognostic model was constructed.

$$GILncSig\;\left(risk\;score\right)=\sum_{i=1}^{n}coef\left(lncRNAi\right)\;\times \;expr\left(lncRNAi\right)$$

In the model, GILncSig was a prognostic risk score for patients with glioma, lncRNAi represented the i-th independent prognostic lncRNA, expr (lncRNAi) was the expression level of lncRNA in the patient, and coef (lncRNAi) represented the contribution of lncRNAi to the prognostic risk score obtained from regression coefficients of multivariate Cox analysis. The median score of patients in the training group was used as a risk cutoff value and used to separate patients into high-risk and low-risk groups (Bao et al., 2020).

### Evaluation of the GILncSig Model

The Kaplan–Meier method and log-rank test were used to evaluate the prognosis of glioma patients under different grouping conditions (Mo et al., 2020). Differences in the instability genes in GILncSig patients (high-risk vs. low-risk) were assessed using the Wilcoxon test. To determine whether GILncSig were independent prognostic factors, univariate and multivariate Cox regression analyses were performed (Li et al., 2019). The performance of GILncSig was evaluated using a time-dependent receiver operating characteristic (ROC) curve (Mandrekar, 2010). The relationships between clinical features and GILncSig were assessed using the Wilcoxon test or Kruskal–Wallis test. Differences in the gene mutation frequency between the high-risk and low-risk groups were assessed using the Chi Square test. Principal component analysis of glioma samples was completed using the scatterplot3d package (Duforet-Frebourg et al., 2016). The correlation circle graph between lncRNAs was drawn using the corrplot package.

### Tumor Immune Microenvironment and GILncSig

Relationships between gene mutations and immune cell infiltration were obtained from the TIMER: Tumor IMmune Estimation Resource website (https://cistrome.shinyapps.io/timer/). We scored 29 immune gene sets in the samples using the single-sample gene set enrichment analysis (GSEA) method, sorted them in an ascending order according to GILncSig, and then separated them into two groups based on the median value (Wang et al., 2020). The abundance of immune cells infiltrating the tumor samples was calculated using the CIBERSORT method (Ostrom et al., 2019).

### Glioma Stemness Index and GILncSig

Differences in stem cell index were determined using the Wilcoxon test and Kruskal–Wallis test for glioma and non-tumor tissue and for different clinical features (Parks, 2018). The tumor samples were divided into a high stem cell index group (high) and low stem cell index group (low) according to the median value. The Kaplan–Meier method and log-rank test were used to evaluate the prognosis of patients with glioma. The correlation between mDNAsi and GILncSig was analyzed according to the Pearson correlation coefficient using GraphPad Prism 7 software. A heatmap related to mDNAsi, GILncSig, tumor microenvironment-related indicators, and mutant genes was drawn using the pheatmap function.

### Gene Ontology (GO) Analysis

Correlation analysis was used to screen for the top 10 mRNAs with the strongest correlation with lncRNAs. These then underwent GO enrichment analysis (Kim et al., 2016).

### Immunotherapy Sensitivity and GILncSig

We obtained scoring data related to immunotherapy efficacy for 152 glioma cases from The Cancer Imaging Archive (TCIA) (https://tcia.at/home; Supplementary Material S1 form). Based on the GILncSig score, we divided the samples into high- and low-risk groups. The relationships between immunotherapy sensitivity and GILncSig were assessed using the Wilcoxon test, and a violin chart was drawn using R. The pRRophetic_0.5 package was used to predict the sensitivity of the high- and low-risk groups to cisplatin and rapamycin chemotherapy drugs.

### Clinical Specimens and Study Ethical Consideration

We collected a total of 30 glioma samples and 5 control samples (post epilepsy specimens) from patients of the First Affiliated Hospital of Harbin Medical University (Harbin, Heilongjiang, China). Consent, including signed informed consent forms, was obtained from family members. The study was approved by the Ethics Committee of The First Affiliated Hospital of Harbin Medical University.

### Quantitative Reverse Transcription-Polymerase Chain Reaction (RT-qPCR) and Immunohistochemistry

The RT-qPCR and immunohistochemistry methods have been previously described (Wang et al., 2020). The sequences of gene primers used in this study are listed in Supplementary Table S1. The primary antibodies against PD-L1 and CTLA4, secondary antibodies, and immunohistochemistry-related kits were all purchased from Affinity Corporation.

### Statistics Software

Strawberry-perl-5.30.2.1, R version 3.6.1, and GraphPad Prism 7 software were used to perform the statistical analyses and graphing. Statistical significance was set at ∗*p* < 0.05, ∗∗*p* < 0.01, and ∗∗∗*p* < 0.001, as indicated.

## Results

### Genome Instability-Related lncRNAs Derived From Somatic Mutations

**FIGURE 1:**
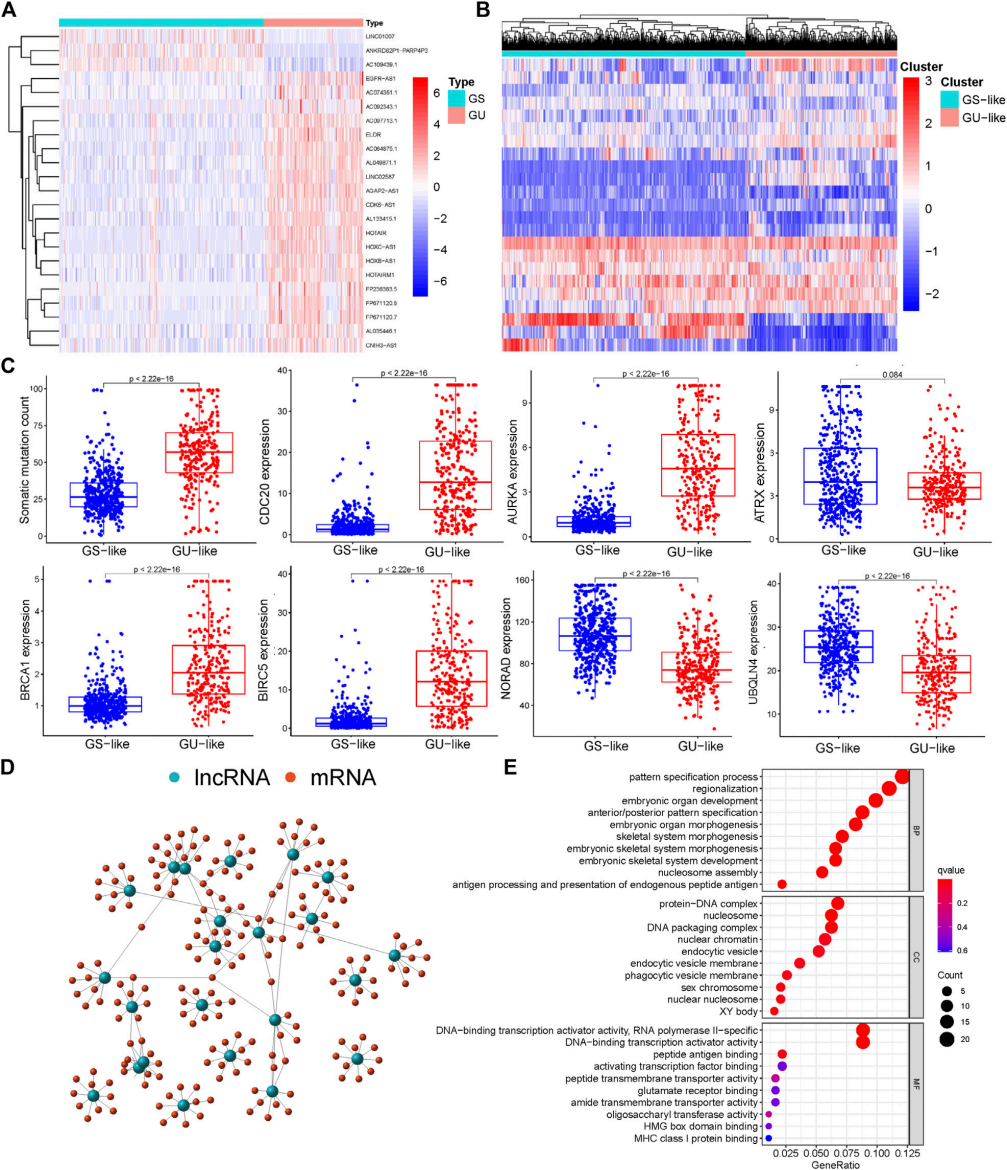
Screening and functional enrichment of genetic instability-related lncRNAs derived from somatic mutations in glioma patients. **(A)** Heatmap of significantly different lncRNAs between genome stability and genome instability samples. The left green cluster is the genome stable (GS)-like group, and the right red cluster is the genome unstable (GU)-like group. **(B)** Unsupervised cluster analysis of 23 lncRNAs related to genome instability in 698 glioma samples. **(C)** Boxplot of differential expression of somatic mutations, CDC20, AURKA, ATRX, BRCA1, BIRC5, NORAD, and UBQLN4, in GS and GU. **(D)** Co-expression analysis of lncRNA and miRNA based on the Pearson correlation coefficient. **(E)** Gene ontology (GO) function enrichment analysis of mRNA co-expressed with lncRNA.

We performed analysis on the differences between the GU-like samples and GS-like samples, identified 23 differentially expressed lncRNAs (Supplementary Table S1), and generated a heatmap to visualize the findings (Figure 1A). Through cluster analysis, all tumor samples were divided into GU-like and GS-like groups; the results revealed that there were significant differences between the two groups (Figure 1B). Quantitative analysis showed that the expression levels of somatic mutations in CDC20, AURKA, BRCA1, and BIRC5 in the GU-like group were significantly higher than those in the GS-like group. In contrast, the expression levels of NORAD, UBXLN4, and ATRX in the GU-like group were significantly lower than those in the GS-like group (Figure 1C). We searched for and identified the top 10 mRNAs exhibiting the strongest correlation with lncRNAs and mapped the co-expression network (Figure 1D). GO analysis revealed that the lncRNAs related to genome instability may have been involved in the malignant behavior of gliomas through protein-DNA complexes, DNA packaging complexes, nucleosome and DNA-binding transcription activator activity, and RNA polymerase II-specific (Figure 1E).

### GILncSig in the Training Group

Using a machine learning model, we randomly divided 667 glioma samples into a training group (*n* = 335) and a test group (*n* = 332). It was determined using the chi-square test that there was no difference among the three data sets, and the experimental results were independently verified (Table 1). Through univariate and multivariate Cox regression analyses, 16 lncRNAs related to prognosis were identified (Supplemental Figure S1) and seven lncRNAs related to independent prognosis were selected from the training group (Table 2).

**TABLE 1:** Clinical information for three Glioma patients groups in this study.

Covariates	Type	TCGA	Test	Train	*P* value
		*n* = 667	*n* = 335	*n* = 332	
Age	≤42	254 (38.54%)	129 (39.09%)	125 (37.99%)	0.6989
	>42	348 (52.81%)	170 (51.52%)	178 (54.1%)	
	unknow	57 (8.65%)	31 (9.39%)	26 (7.9 %)	
WHO	Grade II-III	452 (68.59%)	227 (68.79%)	225 (68.39%)	0.706
	Grade IV	150 (22.76%)	72 (21.82%)	78 (23.71%)	
	unknow	57 (8.65%)	31 (9.39%)	26 (7.9%)	
Gender	Female	252 (38.24%)	124 (37.58%)	128 (38.91%)	0.9128
	Male	350 (53.11%)	175 (53.03%)	175 (53.19%)	
	unknow	57 (8.65%)	31 (9.39%)	26 (7.9%)	
IDH_status	Mutant	421 (63.88%)	215 (65.15%)	206 (62.61%)	0.5124
	unknow	7 (1.06%)	4 (1.21%)	3 (0.91%)	
	Wildtype	231 (35.05%)	111 (33.64%)	120 (36.47%)	
1p19q_status	Codel	167 (25.34%)	76 (23.03%)	91 (27.66%)	0.1709
	Non-codel	488 (74.05%)	254 (76.97%)	234 (71.12%)	
	unknow	4 (0.61%)	0 (0%)	4 (1.22%)	

Chi square test is applied for statistical analysis.

**TABLE 2:** Multivariate Cox regression analyses of the genome instability-related lncRNAs associated with overall survival in Glioma.

Gene symbol	coef	HR	HR.95L	HR.95H	*p* value
HOTAIR	0.176815	1.19341	1.096712298	1.298633	4.11E-05
AC109439.1	−0.43252	0.648874	0.532571848	0.790575	1.77E-05
AL035446.1	0.023758	1.024043	1.003406257	1.045104	0.022176
CDK6-AS1	0.084613	1.088296	1.025776964	1.154626	0.005062
AL133415.1	0.142271	1.152889	1.092884564	1.216188	1.82E-07
HOXC-AS1	−0.09503	0.909346	0.837458986	0.987404	0.023719
AGAP2-AS1	0.009649	1.009695	1.002666019	1.016774	0.006791

Coef, coefficient ; p < 0.05 is considered statistically significant.

**FIGURE 2:**
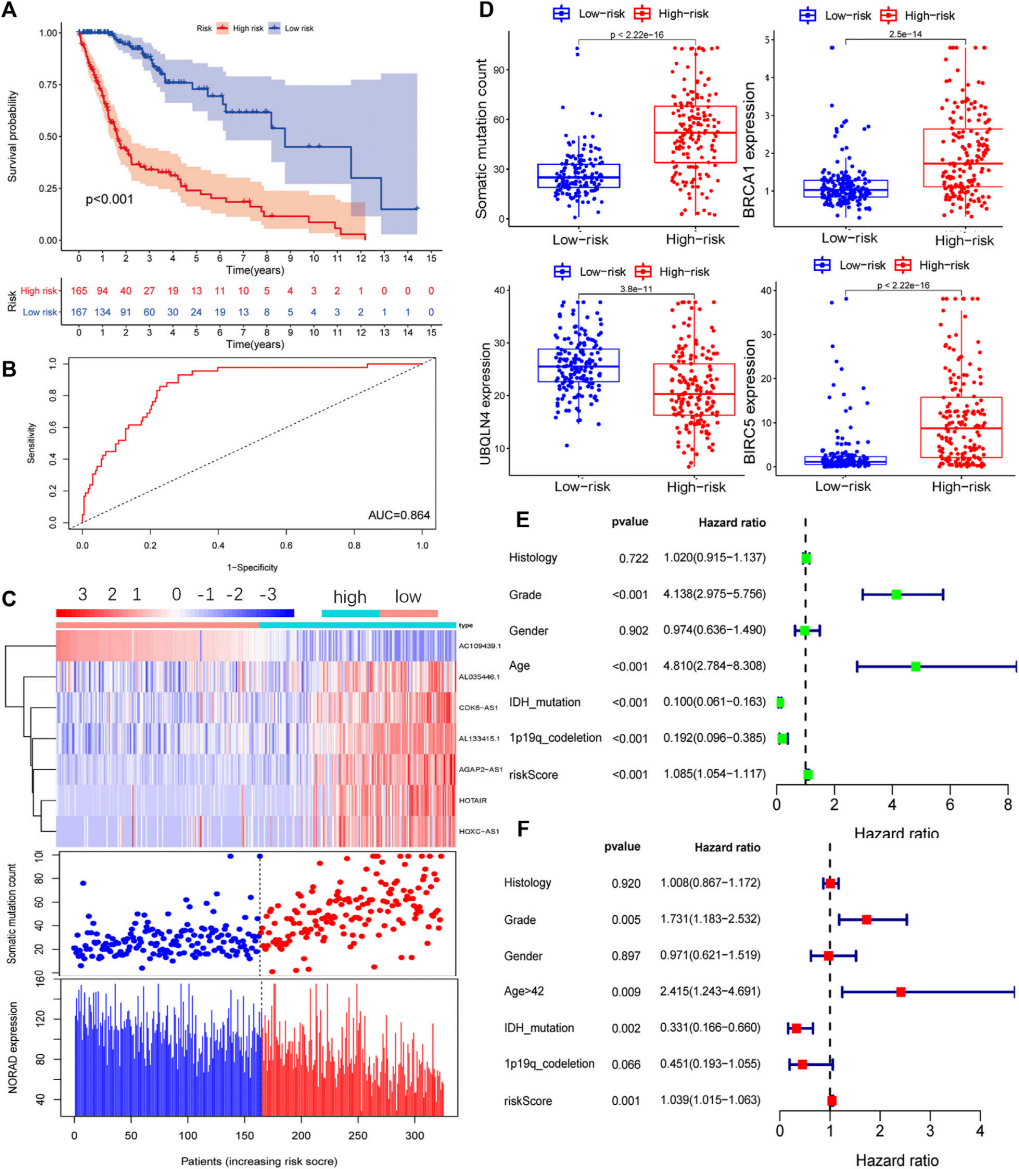
Prognostic model of the genome instability-related lncRNA signature (GILncSig) in the training (Train) group. **(A)** Survival curve of patients based on the GILncSig high- and low-risk groups in the Train group. **(B)** Time-dependent ROC curves analysis of the GILncSig at 1 year. **(C)** LncRNA expression patterns and distribution of somatic mutations and NORAD expression with increasing GILncSig scores. **(D)** Distribution of cumulative somatic mutations, BRCA1, UBQLN4, and BIRC5 expression in the high- and low-risk groups of glioma patients. Statistical analysis was performed using the Mann–Whitney U test. Univariate **(E)** and multivariate **(F)** analyses of the risk score (GILncSig) were related to the prognosis of patients with glioma.

The GILncSig of each sample was calculated according to the formula defined in the Materials and Methods section. The tumor samples were divided into high-risk and low-risk groups, and a survival curve was drawn relative to the median value. In the training group, the prognosis for the high-risk group was found to be worse than that for the low-risk group (Figure 2A). Time-dependent ROC curve analysis of the GILncSig yielded an area under the curve (AUC) of 0.864 (Figure 2B). Ranking the GILncSig from low to high, we determined the expression of AL035446.1, CDK6-AS1, AL133425.1, AGAP2AS, HOTAIR, and HOXCAS; the number of somatic mutations gradually increased, while the expression of AC1094391 and NORAD decreased sequentially (Figure 2C). Quantitative analysis revealed the expression of BIRC5 and BRCA1; somatic mutation values in the high-risk group were higher than those in the low-risk group, whereas UBQLN4 expression was relatively low in the high-risk group (Figure 2D). Univariate analysis revealed that the GILncSig risk score was related to the prognosis of patients with glioma (HR = 1.085, 95% CI = 1.085–1.117, *p* < 0.001; Figure 2E). Multivariate analysis showed that the GILncSig risk score was an independent prognostic factor (HR = 1.039, 95% CI = 1.015–1.063, *p* = 0.001; Figure 2F).

### Independent Validation of GILncSig in Glioma

**FIGURE 3:**
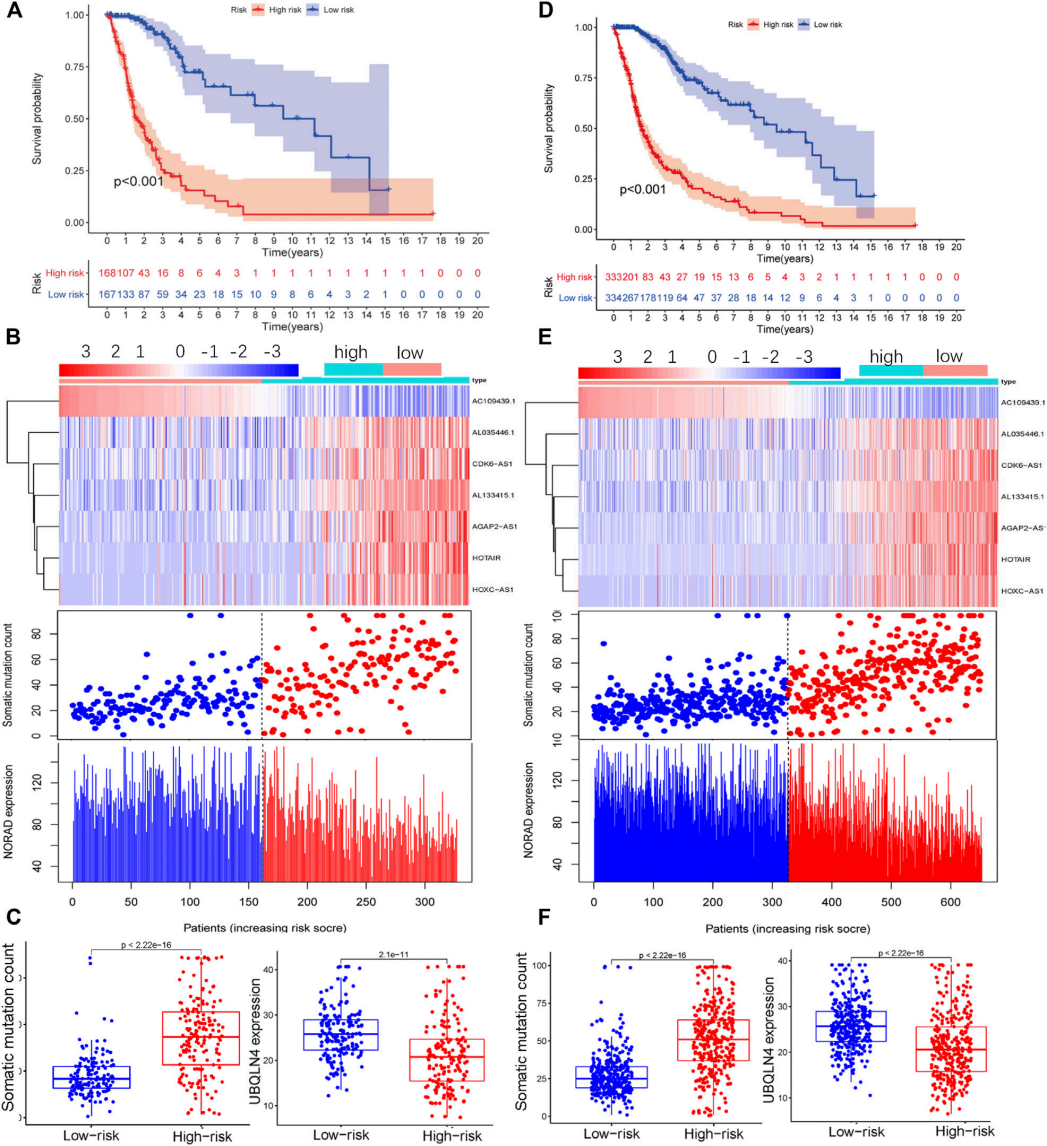
Performance of GILncSig was verified using the test group (Test) and TCGA data. Survival curves of patients in the high- and low-risk groups based on the GILncSig score of the Test group **(A)** and TCGA **(D)** datasets. LncRNA expression patterns and distribution of somatic mutations and NORAD expression with increasing GILncSig scores in the Test group **(B)** and TCGA dataset **(E)**. Distribution of cumulative somatic mutations and UBQLN4 expression in the high- and low-risk groups for glioma based on the Test group **(C)** and TCGA dataset **(F)**.

To confirm the reliability of GILncSig, we conducted independent verification using the test group and TCGA datasets. The lncRNAs and grouping thresholds used to build the model in the training group were applied to these two additional datasets. The Kaplan–Meier method and log-rank test results showed that the prognosis of patients with glioma in the high-risk group was significantly worse than that in the low-risk group (Test, Figure 3A; TCGA, Figure 3D). The expression levels of AL035446.1, CDK6-AS1, AL133425.1, AGAP2-AS, HOTAIR, and HOXC-AS and the number of somatic mutations gradually increased in conjunction with an increase in the expression of GILncSig, while the expression levels of AC1094391 and NORAD decreased (Test, Figure 3B; TCGA, Figure 3E). Further quantification revealed that the somatic mutation value in the high-risk group was higher than that in the low-risk group, while the opposite was true for UBQLN4 (Test, Figure 3C; TCGA, Figure 3F). The time-dependent ROC curve analysis also confirmed that GILncSig had clinical diagnostic value in the test group and TCGA datasets (Test, AUC = 0.881, Supplementary Figure S2A; TCGA, AUC = 0.896, Supplementary Figure S2B). These results were consistent with those for the training group.

### Clinical Features of GILncSig in Glioma

**FIGURE 4:**
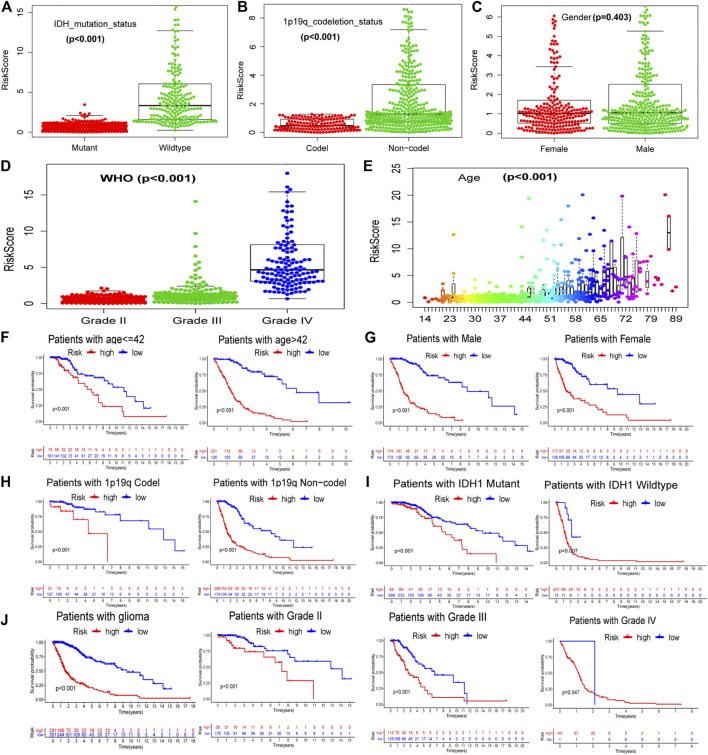
Relationship between GILncSig and clinical prognosis of patients with glioma derived from TCGA. The relationship between GILncSig score (risk score) and clinical characteristics: IDH status **(A)**, 1p19q status **(B)**, gender **(C)**, WHO grade **(D)**, and patient age **(E)**. Survival curves of patients in the high- and low-risk groups based on the GILncSig score under different clinical characteristics: patient age **(F)**, gender **(G)**, 1p19q status **(H)**, IDH status **(I)**, and WHO grade **(J)**.

We extracted the clinical information for glioma samples in TCGA and used statistical tests to analyze the correlation between GILncSig and the clinical features. It was found that the GILncSig risk score in wild-type IDH was higher than that in mutant IDH (Figure 4A), 1p/19q non-codel status was higher than the 1p/19q co-deletion status (Figure 4B), and there was no difference between females and males (Figure 4C). The GILncSig risk score increased with increase in patient age and the World Health Organization (WHO) grade level (Figures 4D,E). We also analyzed GILncSig as a risk factor for the prognosis of patients with different clinical symptoms, except for WHO grade IV (Figures 4F–J). In summary, GILncSig as a marker of patient prognosis was superior to common clinical features.

### Independent Verification of GILncSig Using the CGGA Database

**FIGURE 5:**
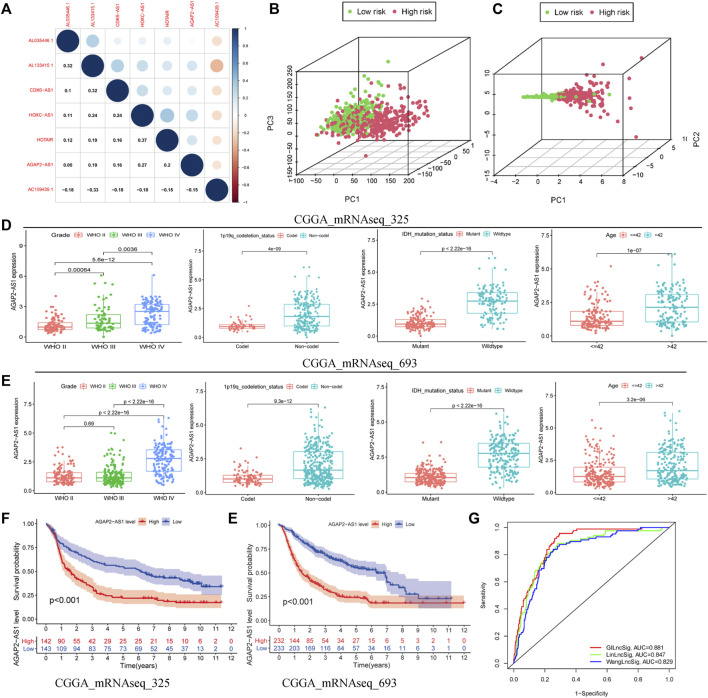
Superiority of the GILncSig model. **(A)** Correlation analysis of lncRNAs derived from GILncSig. **(B)** Principal component analysis based on the expression of all genes. **(C)** Principal component analysis based on the expression of lncRNAs from GILncSig. Clinical features based on one lncRNA (AGAP2-AS1) of GILncSig signature in CGGA-mRNAseq-325 **(D)** and CGGA-mRNAseq-698 **(E)**. Survival curve based on one lncRNA (AGAP2-AS1) of GILncSig in CGGA-mRNAseq-325 **(F)** and CGGA-mRNAseq-698 **(G)**. **(H)** One-year ROC curve based on multiple models.

Correlation analysis revealed that seven independent prognostic-related lncRNAs regulated each other (Figure 5A). Principal component analysis of all the samples revealed that the high-risk and low-risk samples were coalesced with each other (Figure 5B). The model-related linRNA was used as a marker to distinguish the high-risk and low-risk groups (Figure 5C). Of the seven GILncSig lncRNAs in CGGA, we found one lncRNA, that being AGAP2-AS1. Further analysis revealed that the expression of GILncSig increased as the WHO grade level increased. At the same time, the expression levels of GILncSig in the population with 1p19q non-codel, wild-type IDH, and patient age >42 years were higher than those in the population with 1p19q codel, mutant IDH, patient age ≤42 years (CGGA_mRNAseq_325, Figure 5D; CGGA_mRNAseq_693, Figure 5E). The GILncSig high-risk group showed a poor prognosis in the CGGA_mRNAseq_325 (Figure 5E) and CGGA_mRNAseq_693 (Figure 5F) groups. Furthermore, we compared GILncSig, linLncSig, and WangLncSig in all the TCGA samples and found that our model had advantages (Figure 5G; AUC(GILncSig) = 0.881, AUC(LinLncSig) = 0.847, and AUC(WangLncSig) = 0.829). As noted, the GILncSig model exhibited stability and accuracy.

### GILncSig Better Predicted Patient Prognosis Than a Single-Gene Mutation

**FIGURE 6:**
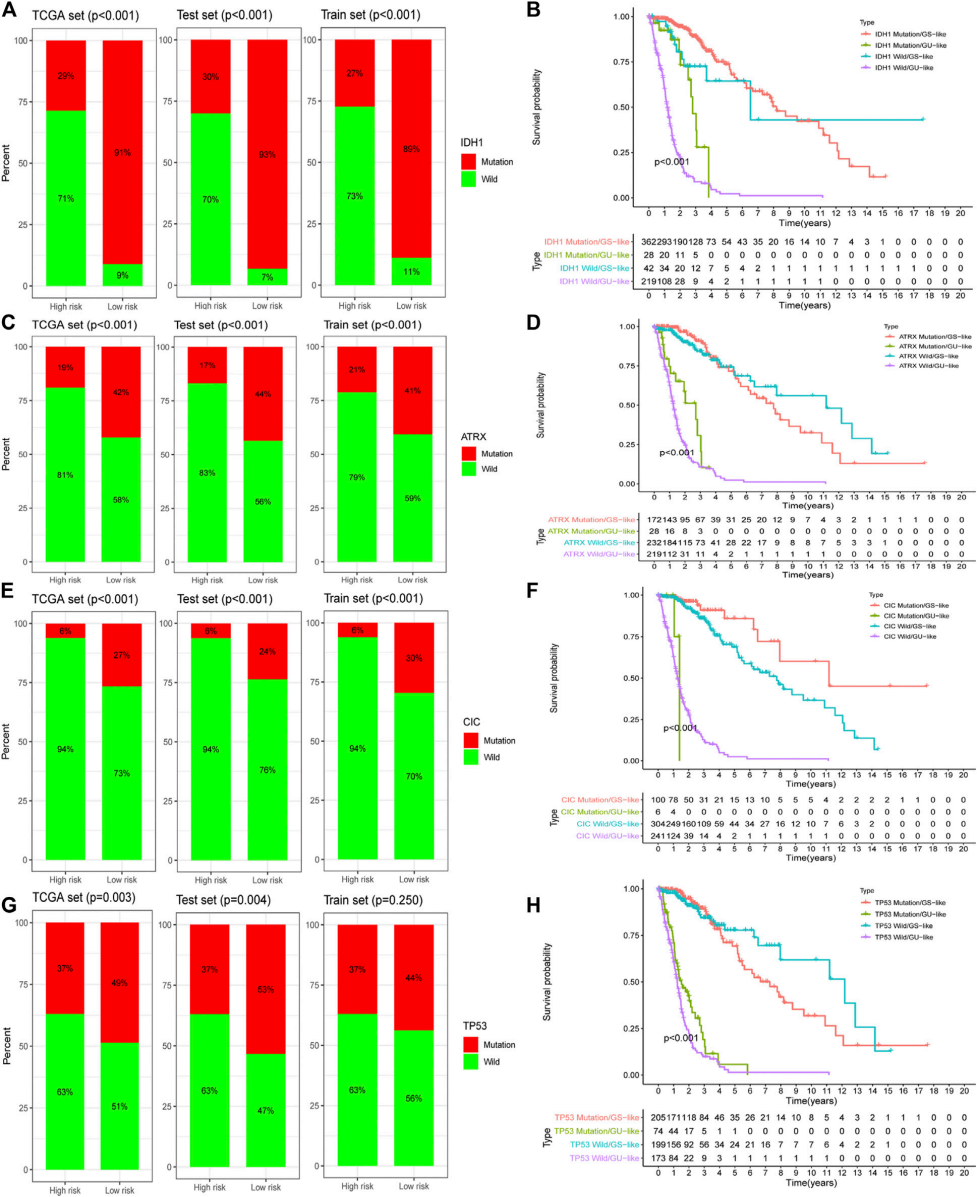
The prognostic assessment ability of GILncSig is better than that of a gene mutation. Proportion of a gene mutation in high- and low-risk groups in the training set, testing set, and TCGA set: IDH1 **(A)**, ATRX **(C)**, CIC **(E)**, and TP53 **(G)**. Survival curves of patients based on the GILncSig scores under different gene mutation states: IDH1 **(B)**, ATRX **(D)**, CIC **(F)**, and TP53 **(H)**.

We screened the most common mutated genes in gliomas, such as *IDH*, *TP53*, *CIC*, and *ATRX*. It was found that variants of these genes made a greater contribution to the genome instability model. We further found that the mutation frequency of these four genes in the low-risk group was higher than that in the high-risk group of the training group, and there were differences between the test group and TCGA dataset (Figures 6A,C,E,G). Survival curve analysis revealed that the prognosis for the GU-like group was much worse than that for the GS-like group in both the mutation group and wild-type group (Figures 6B,D,F,H). GILncSig was better than IDH1, TP53, CIC, and ATRX in determining the prognosis of patients with glioma.

### Tumor Immune Microenvironment and GILncSig

**FIGURE 7:**
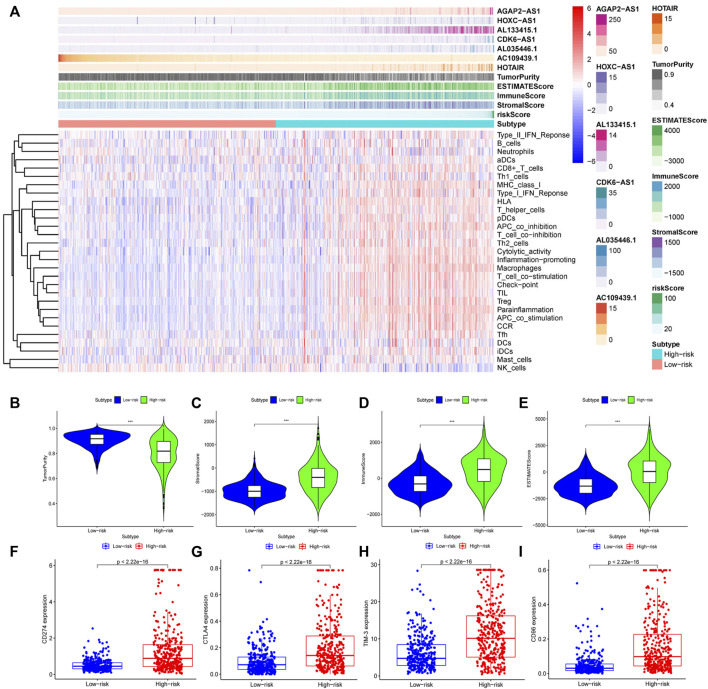
Relationship between GILncSig and the tumor immune microenvironment. **(A)** Heatmap of glioma immune microenvironment based on the GILncSig score. Quantitative analysis of glioma microenvironment and related indicators: tumor purity **(B)**, StromalScore **(C)**, immuneScore **(D)**, and ESTIMATEScore **(E)**. Expression levels of immune checkpoints in the high- and low-risk groups: CD274 **(F)**, CTLA4 **(G)**, TIM-3 **(H)**, and CD96 **(I)**.

**FIGURE 8:**
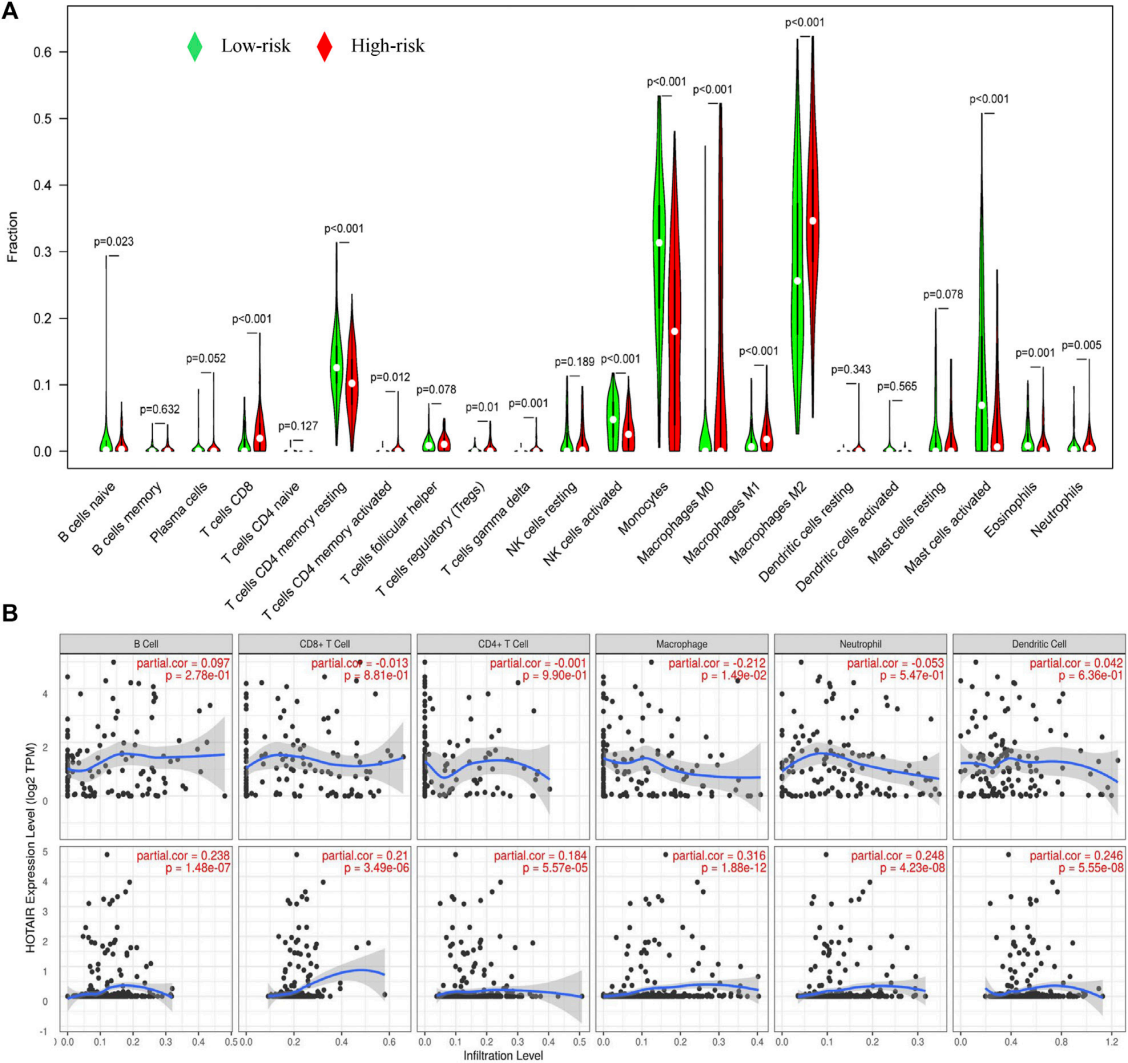
Relationship between GILncSig and immune cell infiltration. **(A)** Distribution of immune cell infiltration in the high- and low-risk groups. Red represents the high-risk group, and green represents the low-risk group. **(B)** Effect of one lncRNA of GILncSig on immune infiltrating cells.

We analyzed the effects of mutated genes on immune infiltration in low-grade glioma (LGG) and glioblastoma multiforme (GBM) and found that in LGG, IDH1 and CIC mutations inhibited the infiltration of six types of immune cells in the glioma microenvironment, while PTEN and ATRX mutations promoted the infiltration of the six immune cell types (Supplementary Figures S3A–S3D). In GBM, PTEN, TTN, and EGFR mutations promoted immune infiltration, while TP53 mutations suppressed immune cell infiltration. However, no statistical significance was found (Supplementary Figures S3E–S3H), perhaps due to the number of GBM samples being relatively small. We concluded that gene mutations are closely related to the immune microenvironment of tumors. Further analysis showed that TumorPurity tended to decrease, while ESTIMATEScore, immuneScore, and StromalScore tended to increase as GILncSig increased (Figure 7A). We also quantified immune microenvironment-related indicators and found that the ESTIMATEScore, immuneScore, and StromalScore values were higher in the high-risk group than in the low-risk group, while TumorPurity was higher in the low-risk group (Figures 7B–E). Expression levels of CD274 (PD-L1), CTLA4, TIM-3, and CD96 in the high-risk group were much higher than those in the low-risk group (Figures 7F–I). We also quantified the immune cells that infiltrated the glioma specimens. Macrophages (M0, M1, and M2) and CD8^+^ T cells infiltrated in greater numbers in the high-risk group than in the low-risk group, while the number of infiltrating naïve B cells, CD4^+^ memory resting T cells, monocytes, activated NK cells, activated mast cells, and neutrophils decreased in the high-risk group compared to that in the low-risk group (Figure 8A). From the TIME website, we found that the expression of one lncRNA of GILncSig, HORAIR, and the infiltration of immune cells were correlated, again proving credibility for our model findings (Figure 8B). We concluded that GILncSig affects the distribution of non-tumor cells in the tumor immune microenvironment and promotes tumor heterogeneity and immunotherapy resistance.

### Tumor mDNAsi and GILncSig

**FIGURE 9:**
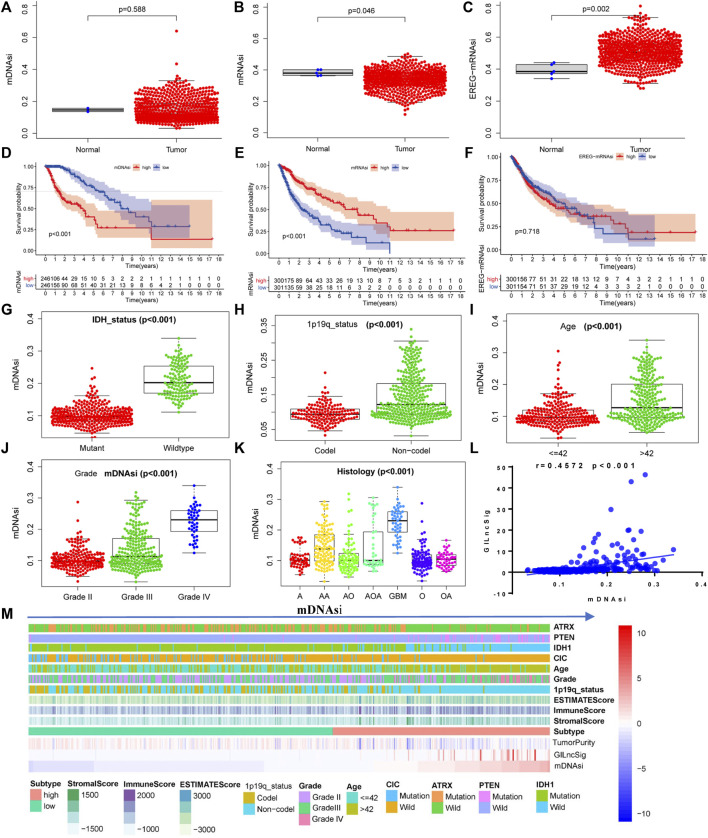
Relationship between the stemness index of glioma cells and GILncSig. Expression of glioma cell stemness indicators in glioma samples and normal tissues: mDNAsi **(A)**, mRNAsi **(B)**, and EGFR-mRNAsi **(C)**. Relationship between the glioma cell stemness index and overall survival of patients: mDNAsi **(D)**, mRNAsi **(E)**, and EGFR-mRNAsi **(F)**. Distribution of the mDNAsi index in patients with different clinical symptoms of glioma: IDH status **(G)**, 1p19q status **(H)**, patient age **(I)**, WHO grade **(J)**, and histology **(K)**. **(L)** Pearson correlation analysis of the GILncSig score and the mDNAsi index. **(M)** Heatmap of glioma immune microenvironment based on the mDNAsi index.

The mean values of mDNAsi and mRNAsi in gliomas were lower than those in normal tissues (Figures 9A,B). In contrast, EGFR mRNA expression in gliomas was higher than that in normal tissues (Figure 9C). Glioma samples were divided into high-expression and low-expression groups according to the mean-based cut-off point. Survival analysis revealed that the prognosis for patients in the mDNAsi high-expression group was worse than that for patients in the low-expression group (Figure 9D), the mRNA high-expression group had better prognosis than the low-expression group (Figure 9E), and there was no significant difference in EGFR mRNA expression between the two groups (Figure 9F, *p* = 0.718). At the same time, mDNAsi expression values were relatively higher for wild-type IDH, 1p19q non-codel, and patient age >42 years than for mutant IDH1, 1p19q codel, and patient age ≤42 years (Figures 9G–I). The mDNAsi expression value also increased as the WHO grade level increased (Figure 9J). Histologically, mDNAsi expression was also relatively high in GBM (Figure 9K). Correlation analysis revealed that the mDNAsi expression value in the sample positively correlated with the GILncSig value (Figure 9l). We also found that the levels of GILncSig in the high-expression group were higher than those in the low-expression group, while the TumorPurity value decreased. At the same time, we found that the values of ESTIMATEScore, immuneScore, and StromalScore increased. In the mDNAsi high-expression group, CIC, IDH1, and ATRX were dominated by wild-type forms, while PTEN was dominated by variants (Figure 9M). We concluded that GILncSig, mDNAsi, and the tumor immune microenvironment were closely related.

### Immunotherapy Sensitivity and GILncSig

**FIGURE 10:**
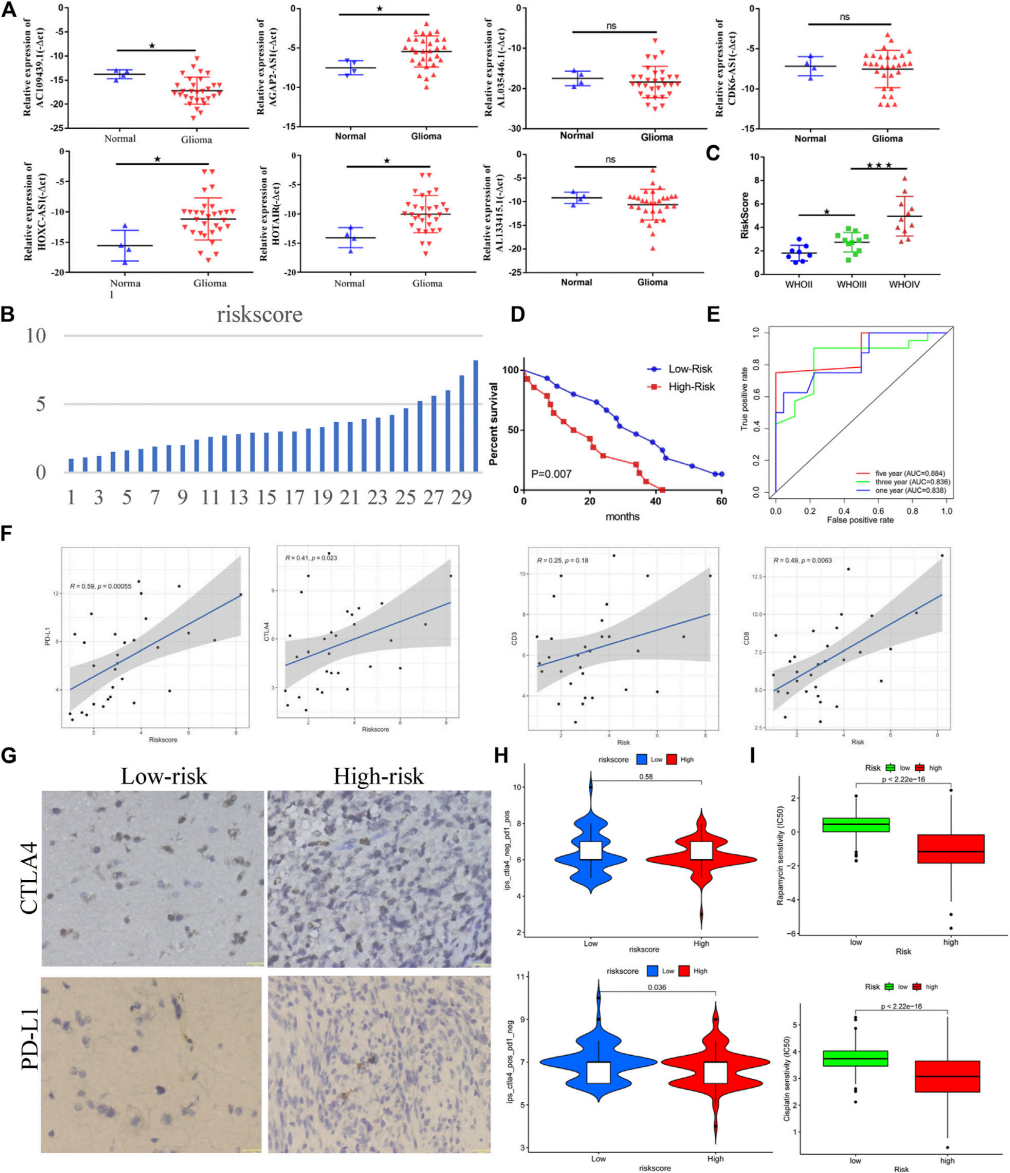
Genome instability-related lncRNAs are closely related to the clinical prognosis and immunotherapy sensitivity of glioma. **(A)** Differential expression of lncRNAs related to genome instability in normal tissue and gliomas. **(B)** Distribution of risk scores of 30 glioma samples. **(C)** Relationship between the WHO classification and risk score (based on lncRNA related to genome instability) in glioma. **(D)** Relationship between the risk score and prognosis of patients with glioma. **(E)** Efficacy of risk score in predicting the prognosis of patients with glioma. **(F)** Correlation between risk score and immune checkpoint of glioma samples. **(G)** Immunohistochemical results of immune checkpoints for the high- and low-risk groups. **(H)** Correlation between the risk score and immunotherapy sensitivity. **(I)** Sensitivity of the high- and low-risk groups to chemotherapy drugs.

Based on the RT-qPCR results, AC109439.1 expression was low, and AGAP2-AS1, HOXC-AS1, and HOTAIR expression was high in glioma samples, while obvious differences in the expression levels of AL133415.1, AL035446.1, and CDK6-AS1 were not observed between glioma and normal tissues (Figure 10A). We calculated the risk scores of 30 glioma samples using the above-noted risk model formula (Figure 10B). Further analysis showed that patient risk scores increased as the WHO grade level of glioma increased (Figure 10C), while the prognosis of patients with high-risk scores was poorer than that of patients in the low-risk groups (Figure 10D). The risk score model demonstrated good performance with regard to predicting the prognosis of glioma patients (Figure 10E, AUC_1years_ = 0.838, AUC_3years_ = 0.836, and AUC_5years_ = 0.884). Correlation analysis revealed that the risk score was closely related to the expression levels of *CD3*, *CD8*, *PD-L1*, and *CTLA4* in glioma patients (Figure 10F). Meanwhile, immunohistochemistry results confirmed that the protein expression of CTLA4 and PD-L1 in the high-risk group was obvious higher than that in the low-risk group (Figure 10G). Patients in the low-risk group showed a better response to anti-CTLA4 treatment than those in the high-risk group, but the anti-PDL1 effect failed to show a significant difference between the two groups (Figure 10H). However, patients in the high-risk group were more sensitive to cisplatin and rapamycin than patients in the low-risk group (Figure 10I).

## Discussion

Based on somatic mutation data, we separated stable genome samples from unstable genome samples. AURKA, CDC20, ATRX, BRCA1, BIRC5, and UBQLN4, which are known to be involved in the process of gene instability (Conde et al., 2017; Sasanuma et al., 2018; Jachimowicz et al., 2019), were used to verify the method. Our results showed that this method could well reflect the characteristics of genome instability. Yosef et al. found that UBQLN4 promotes genome instability and that its high expression indicates a poor prognosis for patients with melanoma (Jachimowicz et al., 2019). Huang et al. found that UBQLN4 can inhibit the proliferation of gastric cancer and that its high expression indicates a better prognosis for patients (Huang et al., 2019). In the current study, we found that as the grade of glioma increased, the expression of UBQLN4 gradually decreased, and its high expression was associated with shorter survival time of patients. Meanwhile, the expression levels of UBQLN4 in the genome instability group were lower than those in the genome stability group. It can be inferred that UBQLN4 is involved in maintaining the stability of the genome in gliomas.

The differentially expressed lncRNAs were screened in the training group and used to construct the GILncSig. Two independent datasets, the test group and TCGA dataset, were used to analyze the correlation between clinical characteristics and prognosis with regard to the model. We found that GILncSig was closely related to both the prognosis and clinical characteristics of patients with gliomas. The model was able to distinguish high-risk and low-risk groups and had certain advantages compared with the previously published models (Lin et al., 2020; Wang et al., 2020). We reviewed the literature and found that AGAP2-AS1, HOTAIR, and HOXC-AS1 (three lncRNAs of GILncSig) are not only related to the malignant progression of tumors and poor prognosis of patients (Tan et al., 2018a; Tan et al., 2018b; Dong et al., 2019) but also affect the tumor immune microenvironment (Wang et al., 2018; Botti et al., 2019). Therefore, clarifying the mechanism of GILncSig in gliomas may provide new ideas for the treatment of gliomas.

Using the TIMER website, we found that gene mutations affect the infiltration of immune cells in the tumor microenvironment. This view is also supported by other investigators (Li et al., 2017a). We speculate that GILncSig is closely related to the tumor immune microenvironment. Our current analysis found the StromalScore, immuneScore, and ESTIMATEScore each increased in the glioma samples as GILncSig expression increased, while TumorPurity tended to decrease. Further evaluation showed that the StromalScore, immuneScore, and ESTIMATEScore were higher in the high-risk group than in the low-risk group, while the TumorPurity score was low in the high-risk group. These findings indicated that the higher the GILncSig expression, the greater the tumor heterogeneity (McGranahan and Swanton, 2017). The expression of PD-L1, CTLA4, TIM-3, and CD96 was significantly higher in high-risk populations than in low-risk populations, indicating that GILncSig expression was related to a high tumor immune resistance (Li et al., 2017b; Saha et al., 2017; Xue et al., 2017; Liu et al., 2020). Macrophage (M0, M1, and M2) and CD8^+^ T-cell infiltration were significantly increased in the high-risk group compared to those in the low-risk group, while the infiltration of naïve B cells, CD4^+^ memory resting T cells, monocytes, activated NK cells, activated mast cells, and neutrophil was significantly reduced. In summary, GILncSig was able to promote tumor heterogeneity and immune resistance by regulating the infiltration and distribution of immune cells in the tumor microenvironment and overexpressing relevant immune checkpoints.

Through laboratory-based experiments, we showed that the expression of immune checkpoints (CTLA4 and PDL1) in the high-risk group was relatively high compared with that in the low-risk group, which was consistent with the above-noted results. Meanwhile, it was also concluded from the TCIA data that patients in the low-risk group were more sensitive to anti-CTLA4 treatment than patients in the high-risk group. Based on the above-mentioned results, we can clearly conclude that patients in the high-risk group were in a state of high immunosuppression. As shown in Figure 7, higher levels of CD8^+^ T-cell infiltration, along with higher levels of macrophage (M0, M1, and M2) infiltration, were observed in the high-risk group. The M1/M2 ratio is typically in a balanced state; a break in this balance may appear as a state of suppressing immunity. The overexpression of CTLA4 and PD-L1 on the surface of CD8^+^ T cells promotes the exhaustion of the CD8^+^ T cells and thereby fails to exert anti-tumor immunity. We plan to use experimental cell biology to verify our assumptions at a later stage. This should provide new insights for the clinical treatment of glioma.

Cancer stem cells have the ability to self-renew, produce heterogeneous tumor cells in tumors, and play an important role in the processes of tumor cell proliferation, invasion, and metastasis (Christensen et al., 2017). The stem cell index describes the degree of acquaintance between tumor cells and stem cells (Vlashi and Pajonk, 2015). We found no significant difference between mRNAsi and mDNAsi in tumor cells compared to those in normal cells. In contrast, EREG-mRNAsi expression was significantly higher in tumor cells than in normal cells. The stemness indicators mRNAsi and mDNAsi are related to patient prognosis and clinical symptoms. We determined that mDNAsi and GILncSig were positively correlated. When the tumor samples were further divided into two groups according to mDNAsi, we found that the tumor samples of the high-expressing mDNAsi group had relatively low TumorPurity, compared with the low expression group, and relatively high StromalScore, immuneScore, and ESTIMATEScore. These results show that the stemness index of glioma cells is closely related to GILncSig expression; additionally, it is also involved in the regulation and distribution of non-tumor cells in the tumor microenvironment, thereby promoting the occurrence of immunotherapy resistance (Nassar and Blanpain, 2016).

Genomic instability is one of the main features of tumor progression and heterogeneity (Hanahan and Weinberg, 2011; Macheret and Halazonetis, 2015; Zhou et al., 2020). Research on its effect has been carried out very early during the process, and some investigators have performed preliminary experimental research. In the current study, we constructed a prognostic model of genomic instability, analyzed its relationship with the tumor microenvironment, and evaluated how genomic instability reduces immunotherapy sensitivity. However, the relationship between lncRNAs and instability-related genes and how lncRNAs are involved in remodeling of the tumor microenvironment remain unclear. We strongly believe that understanding how instability-related genes are involved in malignant progression of glioma may provide novel insights into the clinical treatment of glioma.

## Conclusion

We constructed a framework of genome stability and genome instability based on somatic mutation data to screen for prognostic-related lncRNAs and construct the GILncSig. We verified through multiple platforms that GILncSig was an independent prognostic factor for patients with glioma, was related to the clinical characteristics of the patients, and was able to well distinguish between the high-risk and low-risk groups. In addition, GILncSig positively correlated with the mDNAsi of glioma cells and regulated the infiltration of various immune cells that participate in the remodeling of tumor immune microenvironment cells, such as CD8^+^ T cells and macrophages (M0, M1, and M2). Furthermore, GILncSig was found to be associated with the upregulation of the expression of immune checkpoints, such as CD274, CTLA4, CD96, and TIM-3, which induced tumor immunotherapy resistance. This study provides a new perspective for the clinical treatment of glioma with respect to lncRNAs related to genome instability, tumor immune microenvironment, and glioma cell stemness.

## Data Availability

The datasets presented in this study can be found in online repositories. The names of the repository/repositories and accession number(s) can be found in the article/Supplementary Material.
