# Supplementary figures and images for: Genome Instability-Associated Long Non-Coding RNAs Reveal Biomarkers for Glioma Immunotherapy and Prognosis
Source: Front Genet. 2022 Apr 27;13:850888. doi: 10.3389/fgene.2022.850888 (PMC9094631; doi:10.3389/fgene.2022.850888)

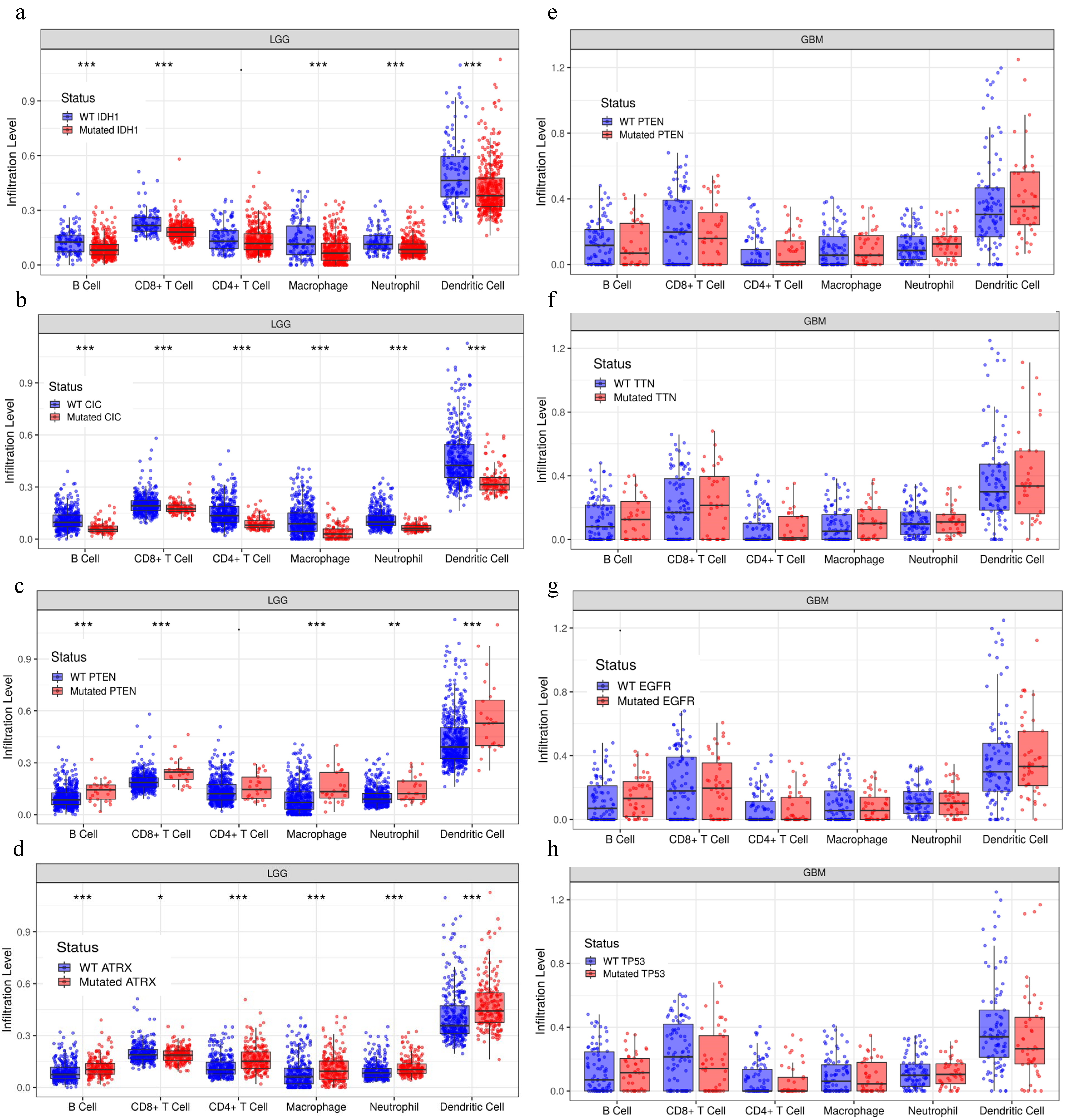

Supplement: Supplementary file 1 [file Image3.TIF]

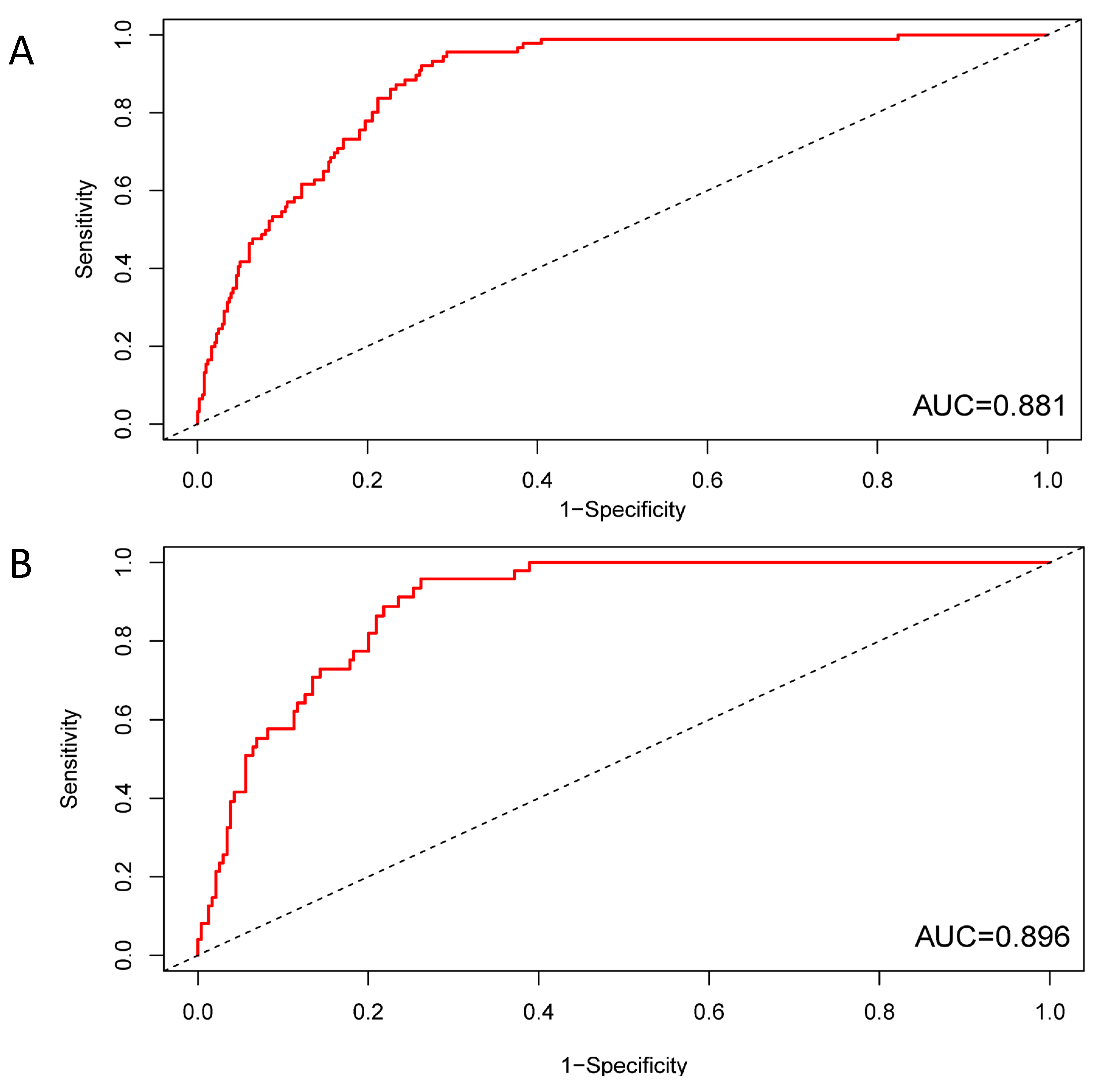

Supplement: Supplementary file 2 [file Image2.TIF]

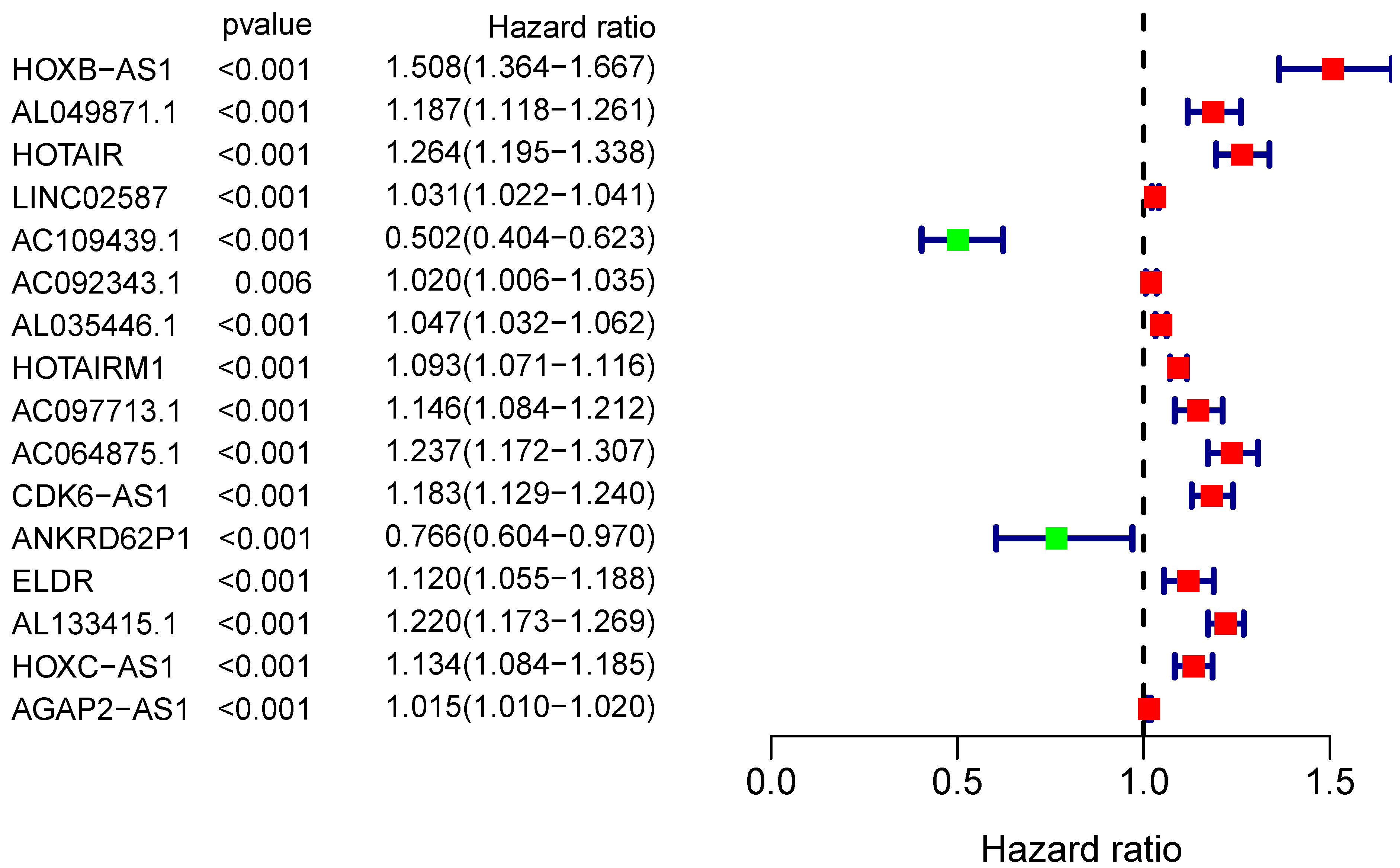

Supplement: Supplementary file 3 [file Image1.TIF]
